# Exploring parent-reported barriers to supporting their child’s health behaviors: a cross-sectional study

**DOI:** 10.1186/s12966-017-0508-9

**Published:** 2017-05-15

**Authors:** Jocelyn W. Jarvis, Daniel W. Harrington, Heather Manson

**Affiliations:** 1 Department of Health Promotion, Chronic Disease and Injury Prevention, Public Health Ontario, 480 University Avenue, Suite 300, Toronto, ON Canada M5G 1V2; 2 School of Public Health and Health Systems, University of Waterloo, 200 University Avenue West, Waterloo, ON Canada N2L 3G1; 3 Dalla Lana School of Public Health, University of Toronto, 155 College Street, 6th floor, Toronto, ON Canada M5T 3M7

**Keywords:** Parental support behaviors, Barriers, Child physical activity, Child recreational screen time, Child healthy eating, Child sleep, Health promotion, Social-ecological framework

## Abstract

**Background:**

Parents can influence the health behaviors of their children by engaging in supportive behaviors (e.g., playing outside with their child, limiting recreational screen time). How, and the extent to which parents engage in supportive behaviors may be influenced by perceived barriers. The purpose of this study is to explore whether the frequency, and types, of barriers to providing parental support are dependent on the type of child health behavior being supported (i.e., physical activity, recreational screen time reduction, healthy eating, and sleep).

**Methods:**

Study participants were 1140 Ontario parents with at least one child under the age of 18 who completed a Computer Assisted Telephone Interview (CATI) survey about parental support behaviors. Open-ended responses about perceived barriers to parental support were coded, and aggregated to meta-categories adopted from the social-ecological model (i.e., individual child, individual parent, interpersonal, environmental). Freidman rank sum tests were used to assess differences across child behaviors. Wilcoxon rank sum tests with Bonferroni adjustments were used as a post hoc test for significant Freidman results.

**Results:**

There were more barriers reported for supporting physical activity than for any other child behavior (*ps* < .01, *As* ≥ .53). Parents reported more parent level and environmental level barriers to supporting child physical activity versus other behaviors (*ps* < .001, *As* ≥ .55), child level barriers were more frequently reported for supporting healthy eating and sleep (*ps* < .001, *As* ≥ .57), and interpersonal barriers were more frequently reported for supporting recreational screen time reduction (*ps* < .001, *As* ≥ .52). Overall, parents reported more child and parent level barriers versus interpersonal and environmental barriers to supporting child health.

**Conclusions:**

Parents experience a variety of barriers to supporting their children’s health behaviors. Differences in types of barriers across child health behaviors emerged; however, some frequently reported barriers (e.g., child preferences) were common across behaviors. Interventions promoting parental support should consider strategies that can accommodate parents’ busy schedules, and relate to activities that children find enjoyable. Creating supportive environments that help facilitate support behaviors, while minimizing parent level barriers, may be of particular benefit. Future research should explore the impact of barriers on parental support behaviors, and effective strategies for overcoming common barriers.

## Background

The physical, mental, social and emotional health of Canadian children and youth is influenced by their participation in several health behaviors. Participating in physical activity [1–3], eating foods that provide a balanced diet rich in fruits and vegetables, and low in sugar-sweetened beverages [4–6], getting enough good quality sleep [7–10], and limiting sedentary recreational screen time [11, 12], all contribute to desirable health outcomes by protecting children against the development of chronic diseases. Concurrently increasing child participation in these distinct behaviors is crucial for improving child health at the population level.

Parents play an important role in their children’s health, in particular, by undertaking parental support behaviors that can influence the extent to which their children engage in health behaviors [13, 14]. For physical activity, for example, this may extend to providing transportation to places where children can be active, participating in physical activity with their children, or discussing the benefits of being active [15–18]. Similarly, setting and enforcing rules about child screen time, and modelling good screen time habits, have been associated with reductions in child screen time [19]. Eating meals together as a family, ensuring healthy foods are easily accessible, and restricting TV-viewing during meals are supportive behaviors and practices associated with child healthy eating [20, 21]. Whereas having a bed time routine and positive parent-child interactions before bed can help to improve child sleep [22]. Supporting each of these child health behaviors is important; however, it is clear that the types of support behaviors (i.e., facilitative, restricting, encouraging) and strategies for support can vary substantially depending on the child behavior being supported, and thus, the desired outcome.

Developing effective interventions to promote parental support behaviors requires an understanding of the barriers to providing that support. A review of interventions targeting parents to improve child weight-related behaviors concluded that identifying barriers parents experience in the early stages of an intervention was a common feature across effective interventions [23]. By identifying barriers to providing support, strategies to overcome these barriers can be developed proactively. A handful of studies and reviews have investigated barriers to parental support for child physical activity, screen time reduction, and/or healthy eating [24–30]. The literature search undertaken for this study yielded no articles that have focused on barriers to supporting child sleep. Results from previous work have identified commonly reported barriers to supporting physical activity as lack of time, safety concerns, child preferences, and weather [24–27, 29, 30]. Common barriers to screen time reduction were child preference for screens, child social networks and communication, parent challenges setting rules and enforcing them due to conflict, weather, parent time, and their own role modelling [26, 28–30]. For healthy eating, child preferences for eating, parent lack of time, cost, parental presence and influence of other carers, and their own role modelling were common barriers [25, 27, 29, 30].

When seeking to understand barriers, it can be useful to group similar types of barriers together using principles from the social-ecological model for health promotion [31]. This model distinguishes influences on behavior change as resulting from intrapersonal (i.e., individual child or parent characteristics), interpersonal (i.e., influences from social networks like friends and family), and broader environmental factors (i.e., community and external influences). This model has been applied previously to understand barriers to supporting child health behaviors [24, 25, 27, 29], with more barriers typically being identified at the child and parent individual levels [29]. This model will be applied in this study to compare and contrast types of barriers across child health behaviors.

Given the breadth of supportive behaviors across distinct child health behaviors, it is reasonable to expect that parents experience diverse barriers to supporting these different child health behaviors. Uncovering both these unique barriers, as well as where similar barriers overlap, is especially relevant as we consider that many current population-level child health programs (e.g., the Healthy Kids Community Challenge [HKCC] in Ontario, Canada; Obesity Prevention and Lifestyle [OPAL] in Australia) as well as new behavioral guidelines (i.e., Canadian 24-Hour Movement Guidelines for Children and Youth [32]) target multiple child health behaviors, and corresponding parental support behaviors, concurrently. Comparing similarities and differences in types of barriers across supporting different child health behaviors may allow for the development of efficient program strategies that can help parents overcome both unique and common barriers to supporting their children. Exploring which child behaviors parents perceive as having the most barriers to support will provide an indication of the areas where parents require the most assistance from program planners and public health.

Building on the literature, the current study aimed to explore whether the number of barriers to providing parental support for child physical activity, recreational screen time reduction, healthy eating, and/or sleep differed by the child health behavior being supported. A secondary objective of this study was to explore the types of barriers parents reported for supporting these individual child health behaviors and whether the types of barriers differed by child health behavior. This study adds to the literature by assessing barriers to supporting different child health behaviors in the same parents, including barriers to supporting child sleep.

## Methods

### Study design

This was a cross-sectional study with a sample of parents living in Ontario, Canada.

### Procedure

This survey was conducted for baseline data collection of a provincial evaluation for a program targeting child physical activity and healthy eating through Ontario communities [33]. This study represents secondary analysis of this baseline survey which focused on parental support behaviors, including barriers to parental support, and parent-reported child health behaviors. Once recruited over the phone, participants provided consent to participate, and responded to the child demographic questions. They were then randomized to complete a child behavior module of questions (i.e., physical activity, screen time, healthy eating, or sleep). Due to the length of the survey, participants were asked to complete at least two behavioral modules; however, they could complete additional optional modules if they agreed. Participants who did not respond to all four behavioral modules of questions were excluded from the analysis. Although parents or guardians (referred to as parents going forward) of children ages less than 1 year were eligible to participate in the survey, they did not complete the behavioral modules and thus were excluded from this analysis. Before concluding the survey, participants were asked to provide demographic information. Data collection for this survey was completed entirely over the phone. This study was approved by the Public Health Ontario Ethics Review Board.

### Participants

Data were collected by a hired research services vendor, between February and March 2015, using Computer Assisted Telephone Interviewing. A random sample of publically available phone numbers was drawn, including both landlines and cellular phones, sampled participants were recruited by phone. Eligible participants were parents with at least one child less than 18 years of age living in their household.

### Measures

#### Demographics

Parents reported their own gender, employment status, household income, marital status, and education. Parents also reported the age and gender of their child with the next birthday, and the number of children living in their household.

#### Barriers to parental support

Barriers to parental support were asked with one open-ended item for each of the child health behaviors being investigated. For child physical activity, healthy eating, and/or sleep participants were asked, “Can you please tell me what, if anything, makes helping your child be (physically active /eat healthy foods/get enough sleep) difficult”? For child screen time participants were asked, “Can you please tell me what, if anything, makes helping your child lower his/her screen time difficult”? An open-ended question was thought to be appropriate to allow unique and broad barriers to be reported; however, common coding categories of potential barriers to parental support were pre-identified before data collection using the literature. Interviewers were instructed to quantitatively indicate responses into the appropriate pre-identified coding category if it emerged. Barriers beyond the pre-identified categories were indicated as “other” and the open-ended response was transcribed verbatim.

### Data analysis

Data analysis was completed using Microsoft Excel 2010 and R version 3.2.3. As the data are non-parametric (i.e., counts), normality was not assessed. Random regression imputation was used to account for missing income data [34]. Specifically, parent age, marital status, employment status, immigration status, and community were used in a prediction model to estimate income values where data were missing.

#### Demographics

Fisher-exact tests for categorical demographic variables, and a Mann-Whitney test for the continuous demographic variable, were used to assess whether there were significant demographic differences between the full participant sample, and the sub-sample included in this study.

#### Coding barriers to parental support

Two coders independently coded 25% of the open-ended “other” barrier responses for each behavior in order to deduce further coding categories, and to code any responses that fit into pre-identified coding categories but were not pre-coded by the interviewers. Overlap of 25% was thought to be conservative and appropriate as the codes reached saturation. The coders then clarified any disagreements regarding the new coding categories that emerged inductively and assigned each code a clarified definition that was added to the codebook for each child behavior. The coders then coded an additional 25% of the open-ended responses for each child behavior category with the finalized codebook. Cohen’s kappa was calculated to establish inter-rater reliability with > .80 being deemed acceptable agreement. Since the interrater reliability exceeded the acceptable agreement (*Cohen’s kappas all > .*80), a single coder completed the remainder of the coding. In addition to the barriers coding categories established prior to data collection, several new codes emerged for each child behavior. In line with previous work, the social-ecological framework [31] was adopted to organize the coding categories by barriers at the individual level (both parent and child), interpersonal level barriers, and environmental barriers.

#### Differences between child health behaviors

Freidman rank sum tests were used to assess differences in the number of reported barriers across child behaviors, and the differences in types (meta-categories) of barriers reported across child behaviors. The frequencies and types of reported barriers represent how often parents’ perceive these barriers, not the relative strength or magnitude of that barrier. Wilcoxon rank sum tests with a Bonferroni adjustment were used as a post hoc test for significant Freidman test results. The Bonferroni adjustment corrects that all effects were reported at a 0.0125 level of significance. Effect sizes were calculated using Vargha and Delaney’s paired *A* statistic, which denotes the probability that a randomly chosen score for one behavior would be greater than a randomly chosen score for the comparator [35, 36]. This effect size does not require parametric assumptions and allows for multiple group comparisons, and can be interpreted as a percent chance of difference, with 0.5 (i.e., 50%) indicating equality between groups [35, 36].

## Results

### Participants

Of the 3206 participants that responded to the survey, 1140 (35.6%) responded to all four behavioral modules and were therefore included in these analyses. Using the American Association for Public Opinion Research Outcome Rate Calculator [37], this study achieved a response rate^1^ of 6.6%, and a cooperation rate^2^ of 11.9%.

Fisher-exact tests for categorical demographic variables revealed that participants who completed all behavioral modules (i.e., subsample) did not significantly differ from the remaining sample by their reported income, marital status, education, gender, gender of child, or number of children in the household (*ps* > .05). The subsample was significantly different from the remaining sample by their language spoken at home, and time since immigration to Canada (*ps* < .05). A Mann-Whitney testfor child age revealed that parents in the subsample had children who were significantly older than the remaining sample (*p* < .05; mean_subsample_ = 9.1 years vs. mean_remaining_ = 8.7 years). Demographic information for the subsample included in the analyses vs. the remaining participant sample is available in Table 1.

**Table 1:** Participant demographics

Demographic characteristic	Subsample [*n* = 1140; % (n)]	Remaining participant sample [*n* = 2066; % (n)]
Parent gender		
Male Female Transgender Don’t know/refused/missing	27.0 (308) 73.0 (832) 0.0 (0) 0.0 (0)	30.1 (622) 69.8 (1442) 0.1 (1) 0.1 (1)
Child gender		
Male Female Don’t know/refused/missing	51.6 (588) 48.4 (552) 0.0 (0)	50.0 (1032) 50.0 (1033) 0.1 (1)
Number of children (<18 years) in household		
1 2 3 4 5+ Don’t know/refused/missing	36.2 (413) 44.6 (508) 14.2 (162) 4.1 (47) 0.9 (10) 0.0 (0)	37.2 (768) 43.9 (906) 14.4 (298) 3.4 (70) 1.1 (23) 0.1 (1)
Age of child with the next birthday*		
Mean age (years)	9.1	8.7
Language at home*		
English French English and French Other Don’t know/refused/missing	85.3 (972) 2.8 (32) 1.4 (16) 10.4 (119) 0.1 (1)	81.0 (1674) 3.0 (62) 1.9 (40) 13.6 (280) 0.5 (10)
Household income		
Less than $30,000 $30,000 to $59,999 $60,000 to $99,999 Greater than $100,000	7.8 (89) 16.2 (185) 27.9 (318) 46.8 (534)	9.8 (202) 15.4 (318) 28.4 (587) 42.9 (887)
Marital status		
Partner No partner Don’t know/refused/missing	83.9 (957) 15.3 (174) 0.8 (9)	85.1 (1759) 14.3 (295) 0.6 (12)
Parent education		
Less than secondary school Secondary school Post-secondary education	2.6 (30) 10.7 (122) 86.1 (982)	3.0 (63) 13.2 (273) 82.8 (1710)
Time since immigration*		
Canadian-born Less than 10 years ago 10 or more years ago Don’t know/Refused/Missing	78.4 (894) 5.4 (61) 15.5 (177) 0.7 (8)	73.2 (1513) 7.1 (147) 17.2 (356) 2.4 (50)

* = significant difference of *p* < .05

Note. Subsample is parents who responded to all four behavioral modules, and were subsequently included in analyses

### Barriers coding

Descriptive statistics for the number of barriers to parental support reported by each parent by child health behavior are presented in Table 2. In general, the majority of parents reported at least one barrier to supporting each child health behavior, with less than a third reporting no barriers.

**Table 2:** Number of barriers to support reported by each participant by child health behavior

Reported barriers per participant (#)	Barriers to supporting child physical activity (%(n))	Barriers to supporting child screen time reduction (%(n))	Barriers to supporting child healthy eating (%(n))	Barriers to supporting child sleep (%(n))
None	26.9 (307)	30.5 (348)	27.3 (312)	29.7 (339)
1	49.8 (568)	50.5 (576)	55.5 (633)	54.5 (621)
2	17.7 (202)	14.7 (168)	13.5 (154)	12.6 (144)
3	4.4 (50)	3.6 (41)	2.2 (25)	2.7 (31)
4	0.9 (10)	0.4 (4)	1.3 (15)	0.4 (4)
5 or more	0.3 (3)	0.3 (3)	0.1 (1)	0.1 (1)

The number of coding categories for barriers to supporting each child health behavior were 30, 36, 25, and 32 for child physical activity, recreational screen time, healthy eating, and sleep respectively. Descriptive statistics for the number of parent reported barriers by child health behavior, by both coding categories and meta-categories from the social-ecological model, are available in Table 3.

**Table 3:** Descriptive statistics of reported barriers to parental support by coding categories and meta-categories

Barrier meta-category	Barrier coding category	Reported barriers (*n* = 4351)				
		Total	Supporting child physical activity	Supporting child screen time reduction	Supporting child healthy eating	Supporting child sleep
Child individual factors	Child preferences	955	108	233	419	195
	Child's age	305	67	77	75	86
	Child's schedule	207	54	6	12	135
	Child health/medical	193	71	15	32	75
	Special/favorite episodes	43	0	43	0	0
	School and homework	31	11	0	0	20
	Pressure/stress	28	0	0	0	28
	Food allergies	15	1	0	14	0
	Child confidence	15	11	0	0	4
	What child eats	14	0	0	0	14
	Getting enough sleep	12	6	1	0	5
	Habit	1	0	1	0	0
	Total	1819	329	376	552	562
Parent individual factors	Time/busy	499	228	97	137	37
	Lack of parental control	252	28	103	55	66
	Work schedule	215	96	40	42	37
	Cost	172	97	6	63	6
	Lack of willpower	68	20	33	8	7
	Inconvenience	50	16	6	23	5
	Lack of alternate activity	44	0	44	0	0
	Lack of routine	34	0	0	0	34
	Parent health/medical	29	25	4	0	0
	Skills required	25	6	7	10	2
	Need for distraction	22	0	22	0	0
	Hard to keep up with expert recommendations	6	0	2	3	1
	Convenience	4	0	4	0	0
	Reward	2	0	2	0	0
	Total	1422	516	370	341	195
Interpersonal factors	Friends or family's perceptions	100	8	60	23	9
	Peer influence	88	9	34	30	15
	Social activity/communication	58	5	39	4	10
	Parental presence	56	4	17	28	7
	Special occasions	43	2	2	6	33
	Family distraction	38	0	0	0	38
	Sibling influence	31	0	9	4	18
	Parent role modelling	30	4	9	11	6
	Peer availability	29	15	14	0	0
	Siblings	17	4	6	0	7
	Social norm	10	0	10	0	0
	Family purchasing behavior	5	0	0	5	0
	Family routine	2	0	2	0	0
	Total	507	51	202	111	143
Environmental factors	Weather	244	172	72	0	0
	Limited access to goods or services	98	66	9	22	1
	Availability of electronics	80	18	17	0	45
	Household sleep environment	20	0	0	0	20
	Availability of alternatives	17	0	0	17	0
	Neighborhood safety	13	13	0	0	0
	Media/advertising	13	0	1	12	0
	External sleep environment	5	0	0	0	5
	Geography	1	1	0	0	0
	Total	491	270	99	51	71
Other	Total	112	15	20	26	51

### Differences between child health behaviors being supported

#### Number of parent-reported barriers by child health behavior

The number of parent-reported barriers to parental support were significantly different when considering the different child health behaviors being supported (χ^2^(3) = 22.31, *p* < .001). Post hoc tests revealed that there were significantly more barriers reported for supporting child physical activity compared to supporting recreational screen time reduction (*V* = 99967, *p* < .001, *A* = .53) healthy eating (*V* = 101830, *p* = .002, *A* = .54) and sleep (*V* = 107130, *p* < .001, *A* = .55). There were no other differences between behaviors (*ps* > .0125, .48 ≤ *As* ≥ .52).

#### Types of parent-reported barriers by child health behavior

*Child individual level barriers* were significantly different by child health behavior being supported (χ^2^(3) = 151.46, *p* < .001). Post hoc tests revealed that compared to physical activity, there were significantly more child barriers reported to supporting both child healthy eating (*V* = 29316, *p* < .001, *A* = .59) and sleep (*V* = 35396, *p* < .001, *A* = .59). Similarly, compared to recreational screen time there were significantly more child barriers reported to supporting both child healthy eating (*V* = 42202, *p* < .001, *A* = .58) and sleep (*V* = 36318, *p* < .001, *A* = .57). There were no other differences between behaviors (*ps* > .0125, .50 ≤ *As* ≥ .52).

*Parent individual level barriers* were significantly different by child health behavior being supported (χ^2^(3) = 159.73, *p* < .001). Post hoc tests revealed that there were significantly more parent level barriers to supporting child physical activity (*V* = 80198, *p* < .001, *A =* .61), recreational screen time reduction, (*V* = 46670, *p* < .001, *A =* .56), and healthy eating (*V* = 44472, *p* = .004, *A =* .55), compared to supporting sleep. In addition, there were more parent level barriers to supporting child physical activity than supporting recreational screen time reduction (*V* = 63260, *p* < .001, *A* = .55) or healthy eating (*V* = 68214, *p* < .001, *A* = .56). There were no other differences between behaviors (*p* > .0125, *A* = .51).

*Interpersonal barriers* were significantly different by child health behaviors being supported (χ^2^(3) =111.34, *p* < .001). Post hoc tests revealed that there were significantly more interpersonal barriers identified for supporting child recreational screen time reduction compared to supporting physical activity, (*V* = 3387, *p* < .001, *A =* .56), healthy eating, (*V* = 18572, *p* < .001, *A =* .54), or sleep, (*V* = 20271, *p* < .001, *A =* .52). In addition, compared to physical activity, there were significantly more interpersonal barriers reported to supporting both child healthy eating (*V* = 2546, *p* < .001, *A =* .53) and sleep (*V* = 3143.5, *p* < .001, *A =* .54). There were no other differences between groups (*p* > .0125, *A =* .52).

*Environmental barriers* were significantly different by child health behavior being supported (χ^2^(3) = 253.47, *p* < .001). Post hoc tests revealed that environmental barriers were reported significantly more frequently for supporting child physical activity compared to recreational screen time reduction (*V* = 28027, *p* < .001, *A =* .57), healthy eating (*V* = 29118, *p* < .001, *A =* .59), and sleep (*V* = 31330, *p* < .001, *A =* .58). In addition, compared to healthy eating, parents reported significantly more environmental barriers to supporting both child recreational screen time reduction (*V* = 6278.5, *p* < .001, *A =* .52) and sleep (*V* = 1925, *p* < .001, *A =* .51). There were no other differences between groups (*p* > .0125, *A =* .51).

## Discussion

The results for both the number of parent-reported barriers to engaging in parental support activities, as well as the types of barriers reported, yielded several important and interesting findings. Though effect sizes were small, parents reported significantly more barriers to supporting their child’s physical activity than any other child health behavior.

Considering types of barriers reported in the context of the social-ecological model, significant differences emerged by child behavior. At the individual level, parents reported more child barriers to supporting child healthy eating and sleep than either physical activity participation or recreational screen time reduction. Conversely, more parent level individual barriers were reported for supporting child physical activity participation than any of the other child health behavior. More parent level barriers emerged for supporting both healthy eating and recreational screen time reduction than for supporting sleep, with small effect sizes. When considering barriers related to the interpersonal influence of others, parents reported significantly more barriers to supporting child recreational screen time reduction than any other child behavior, with healthy eating and sleep support barriers being reported more often than physical activity support barriers, all with small effect sizes. Similar to the parent level barriers, parents reported significantly more environmental type barriers to supporting child physical activity participation than any other child health behavior. More environmental barriers were also reported for supporting recreational screen time reduction and sleep than for supporting healthy eating, with small effect sizes.

For child physical activity, parents reported experiencing more barriers in general, and more parent and environmental level barriers specifically, than for supporting any other child behavior. The difference in the number of barriers being reported may be understood by comparing the types of barriers, and types of support behaviors, involved with supporting physical activity versus the other behaviors. Many types of support for child physical activity involve direct and active participation from parents (e.g., play outside with them), whereas for other child behaviors parental support may be more restrictive (e.g., setting rules for screens and bed time) or efficient (e.g., making dinner for the whole family at once, purchasing healthier options). As such, physical activity support behaviors may be more effortful and time-consuming for parents in addition to their everyday tasks, presenting more barriers. Common parent level (e.g., time/busy), and environmental barriers (e.g., weather) to supporting child physical activity that were identified in this study are also common in the literature [24–27]. The prevalence of these reported barriers suggests that they may be barriers that parents have difficulty overcoming. This makes sense given parent work commitments, (e.g., “shift work taking away all my family time”) and the unpredictable nature of the weather, (e.g., “freezing temperatures”), might be realistically or perceived to be outside of parents’ control. Interestingly, time as a barrier may be due to competing demands of parents, or it may be reflective of parents valuing other activities for their children above physical activity, such as homework [24]. Strategies that can help parents support their children’s physical activity within limited time constraints (e.g., getting children to assist with active chores, letting them walk/ride a bike to school, registering them for active after-school programs in the community), and in a variety of weather scenarios (e.g., winter, rain), may be particularly beneficial for improving parental support for child physical activity.

Parents reported experiencing more interpersonal barriers to supporting child recreational screen time reduction that any other type of behavior, although the effect size was small. This is not surprising given that screens are commonly used by children as a means of communicating with their social networks [26, 28], and are increasingly being integrated into society [29]. When observing common barriers (of any type) to supporting recreational screen time, at the parent level lack of parental control (e.g., “he/she is so strong willed” or “he/she does not listen”), and a lack of alternate activity (e.g., “finding something else that he/she can do herself”), were prominent whereas at the child level child preferences (e.g., “he/she is obsessed”, “they enjoy screen time”) was common, aligning with previous studies [26, 28, 29]. Since children both enjoy screen time and use it for peer communication, parents may be met with resistance when trying to support screen time reduction [28]. This may lead parents to feel a lack of control over their child’s behavior. However, finding alternate activities that children enjoy as much as screen time, that provide other opportunities for peer interaction, and that do not require active investment from parents and thereby manifest in other barriers (e.g., time/busy), may be challenging.

Child level barriers to supporting healthy eating and sleep were reported more often than for physical activity or recreational screen time. Interestingly, child level barriers for healthy eating and sleep accounted for the two most reported categories of barriers at any social-ecological level for any behavior. Taken together, it is evident that not only are more child level barriers being reported for healthy eating and sleep in comparison to other child behaviors, but that many parents are experiencing these types of barriers. For healthy eating, the vast majority (75%) of these barriers were accounted for by child preferences (e.g., “does not like certain fruits or veggies” or “the taste and texture of some foods”), whereas for sleep the majority was split between child preferences (35%; e.g., “doesn’t want to go to sleep”) and child schedule (24%; e.g., “evening activities” or “if he/she had a nap during the day”). Similar to recreational screen time, child preferences as a barrier to supporting healthy eating is well documented in the literature [25, 27, 29]. Assisting parents in developing the food skills and confidence to prepare foods that are both healthy and that children enjoy is a strategy worthy of further investigation. Although literature relating to supporting child sleep was not found, the barrier of child preferences emerged also for sleep indicating that strategies that make bed time more enjoyable for children may be effective.

It is possible that these potentially useful strategies (i.e., find alternate activities more enjoyable than screen time, make foods healthy and enjoyable, and make bed time fun), which shift the type of parental support behaviors from more restrictive behaviors, to facilitative and participatory (similar to physical activity), might amplify other parent level barriers such as parent time, busy schedules, and cost. This raises an important consideration about achieving efficiencies in feasible ways. That is, what strategies make sense to combat common barriers? Will these ultimately be feasible for parents to implement in the face of new or evolving barriers? The burden that the aforementioned strategies for support place on parents is especially relevant considering that parents already report more barriers at the parent and child individual levels than interpersonal and environmental levels [29]. As such, alleviating child level barriers with strategies that may manifest in additional parent level barriers to support, might not be the most effective approach for reducing the overall number of barriers, and ultimately increasing parental support and child health. Although parental support strategies should make suggestions to parents about how they can effectively support their child within their given constraints, perhaps strategies focusing on creating supportive environments, thereby making it easier and more feasible for parents to engage in support behaviors, might be a more effective use of resources.

This study is not without limitations. These analyses were conducted in a subsample of parents that decided to optionally complete two additional behavioral modules of a study. It is possible that this subsample represents a subpopulation of parents that are interested in child health behaviors. In this survey, parents were asked separately about whether they experienced barriers to accessing sports and recreation facilities. As such, some parents might have under-reported limited access to goods and services barriers for supporting child physical activity (felt they had already reported), or over-reported those barriers (already primed to consider them). Lastly, this study analyzed barriers to parental support considering the number of barriers that parents’ reported, taking a greater number of barriers to mean that it was more often experienced by parents. However, we did not collect data regarding the magnitude of importance of each barrier, and therefore lack information about how impactful these barriers are to inhibiting parental support. Future research should extend these findings by seeking to understand which of the most commonly reported barriers identified here are most debilitating to parental support, and for what reasons. In doing so, it can be determined which specific barriers are both prevalent and challenging for parents to overcome, and thus make meaningful targets for interventions promoting parental support.

Despite these limitations, this study presents several strengths. Specifically, this study extends qualitative work on barriers to parental support by quantifying which types of barriers are reported most frequently by parents across unique child behaviors. Using repeated measures to assess barriers to support for four distinct child behaviors in the same sample of parents is novel and allowed for complex comparison and interpretation between behaviors. Lastly, there is a lack of literature on barriers to parental support for child sleep. Given the growth of research on the importance of child sleep to health, acknowledged in the literature as well as by the inclusion of sleep in the most recent Canadian 24-Hour Movement Guidelines for Children and Youth [32], understanding how parents can support their child in getting enough sleep is a health promotion priority. This study can be considered a first step in beginning to understand which types of barriers to parental support might exist for sleep.

## Conclusions

Parents reported different numbers of barriers to supporting child physical activity, recreational screen time, healthy eating, or sleep, with physical activity having marginally more reported barriers. Parents also reported experiencing different types of barriers when supporting these distinct child health behaviors. Overall, parental support strategies that help parents overcome the constraints of a busy schedule for supporting physical activity, and child level barriers such as child preferences are needed. Importantly, these strategies cannot undermine the fact that asking parents to *do more*, might not be feasible. Further research investigating which barriers are most challenging for parents to overcome, and why, and the effectiveness of strategies to overcome these common and challenging barriers, is needed.

## Footnotes

1 Number of people who participated, divided by the total number of eligible people in the total sample

2 Number of people who participated, divided by the number of eligible people with whom contact was made
